# Feasibility of delivering integrated COPD-asthma care at primary and secondary level public healthcare facilities in Pakistan: a process evaluation

**DOI:** 10.3399/bjgpopen18X101632

**Published:** 2019-02-20

**Authors:** Muhammad Amir Khan, Muhammad Ahmar Khan, John D Walley, Nida Khan, Faisal Imtiaz Sheikh, Saima Ali, Ehsan Salahuddin, Rebecca King, Shaheer Ellahi Khan, Farooq Manzoor, Haroon Jehangir Khan

**Affiliations:** 1 Chief Coordinating Professional, Association for Social Development, Islamabad, Pakistan; 2 Research Coordinator, Association for Social Development, Islamabad, Pakistan; 3 Professor of International Public Health, Nuffield Centre for International Health and Development, University of Leeds, Leeds, UK; 4 Project Coordinator, Association for Social Development, Islamabad, Pakistan; 5 Research Coordinator, Association for Social Development, Islamabad, Pakistan; 6 Research Coordinator, Association for Social Development, Islamabad, Pakistan; 7 Research Coordinator, Association for Social Development, Islamabad, Pakistan; 8 Lecturer, Nuffield Centre for International Health and Development, University of Leeds, Leeds, UK; 9 Provincial Manager, Non-Communicable Disease Control Program, Punjab, Pakistan; 10 Assistant Professor, Humanities and Social Sciences Department, Bahria University, Islamabad, Pakistan; 11 Director, NCD & Mental Health, Directorate General of Health Services, Lahore, Pakistan

**Keywords:** Integrated care, public health facilities, mixed method research, Asthma, COPD

## Abstract

**Background:**

In Pakistan,the estimated prevalence of chronic obstructive pulmonary disease (COPD) and asthma are 2.1% and 4.3% respectively, and existing care is grossly lacking both in coverage and quality. An integrated approach is recommended for delivering COPD and asthma care at public health facilities.

**Aim:**

To understand how an integrated care package was experienced by care providers and patients, and to inform modifications prior to scaling up.

**Design & setting:**

The mixed-methods study was conducted as part of cluster randomised trials on integrated COPD and asthma care at 30 public health facilities.

**Method:**

The care practices were assessed by analysing the clinical records of *n* = 451 asthma and *n* = 313 COPD patients. Semi-structured interviews with service providers and patients were used to understand their care experiences. A framework approach was applied to analyse and interpret qualitative data.

**Results:**

Utilisation of public health facilities for chronic lung conditions was low, mainly because of the non-availability of inhalers. When diagnosed, around two-thirds (69%) of male and more than half (55%) of female patients had severe airway obstruction. The practice of prescribing inhalers differed between intervention and control arms. Patient non-adherence to follow-up visits remained a major treatment challenge (though attrition was lower and slower in the intervention arm). Around half of the male responders who smoked at baseline reported having quit smoking.

**Conclusion:**

The integrated care of chronic lung conditions at public health facilities is feasible and leads to improved diagnosis and treatment in a low-income country setting. The authors recommend scaling of the intervention with continued implementation research, especially on improving patient adherence to treatment.

## How this fits in

Integrated care of chronic lung conditions is a known priority of non-communicable disease programmes, and evidence is needed for an informed programme scaling decision. This process evaluation study was conducted, as part of two intervention trials, to help the programme understand and refine the delivery and support of COPD and asthma care in public health facility settings.

## Introduction

The global prevalences of COPD and asthma were estimated at 251 million and 235 million respectively, causing 3.17 million and 383 000 annual deaths.^1,2^ Pakistan, a lower-middle income country with a population of around 200 million, is facing a high burden of chronic respiratory diseases, including COPD and asthma. The age-standardised mortality rate due to chronic respiratory diseases is estimated to be 138.2 per 100 000 in males and 41.3 per 100 000 among females in Pakistan. A multi-country survey (BREATHE) reported a 2.1% prevalence of COPD among Pakistani adults aged >40 years. It is expected that the COPD morbidity and mortality rates will increase in concurrence with the increasing trend of smoking, one of its major risk factors. According to a national health survey, prevalence of smoking was 28.6% among males and 3.4% among females.^3^ Clinical asthma prevalence has been estimated to be 4.3%,^4^ and as high as 10.7% among children aged 13–14 years.^5^ High chronic respiratory disease prevalence poses a challenge to economic development, and to the quality of life of the population. Integration of clinical management at primary health care (PHC) level is the recommended approach to achieve universal coverage of non-communicable disease care.^6^

In Pakistan, PHC is delivered through a network of PHC facilities (such as basic health units at union council, and rural health centres at cluster levels)^7^ and sub-district level hospitals. The District Health Office is responsible for managing the provision of public-funded care at primary and secondary level facilities. These publicly-funded health centres and hospitals are staffed with doctors and allied staff, and are equipped with basic laboratory facilities such as blood and urine testing, and microscopy. The lack of programme guidelines and tools for diagnosis, treatment, and education of patients is known to result in a widely varied and poor quality care provision, as well as inadequate patient response to the care requirements.^8^

To address the practice variation and care quality challenges, a set of contextualised products were developed for delivering integrated chronic lung care at primary and secondary level facilities. The Provincial Non-Communicable Disease Programme and its partners developed the contextualised products by adapting international best practice guidelines.^9–11^ The care delivery products mainly included: a case management desk-guide; training for doctors and allied staff; and an education tool for patients (Box 1).

**Box 1.** Logic model of an integrated intervention

Intervention inputs	Intervention process and actions	Intended		
		Process change	Outputs	Health outcomes
Case management desk-guide & counselling tool Training of doctors and allied staff (on full care package) Supplement drugs and supplies (for example, peak flow meter) Recording forms^a^	Screen and diagnose^a^ Prescribe and/or dispense asthma and/or COPD drugs Counsel on lung condition; also smoking cessation (if applicable) Follow up care, including retrieval	Providers practise programme protocols to: screen, diagnose, treat, counsel, follow up, and report as per programme protocol Patients: attend follow-up visits adhere to treatment cease smoking (if applicable)	Patient are: screened and diagnosed as per programme protocol prescribed and/or dispensed correct drug and dose counselled for smoking cessation followed-up and treated for continued care	Asthma control and BODE index change in COPD patients

^a^These inputs and practices for screening, diagnosis, and recording were the same in intervention and control arms to ensure comparing ‘like with like’ asthma and COPD patients.

BODE index = Body-mass index, airflow Obstruction, Dyspnea, and Exercise index. COPD = chronic obstructive pulmonary disease.

During the intervention development (through the Technical Working Group of stakeholders) certain assumptions were made about the circumstances and behaviour of health facility staff and patients with chronic respiratory disease. A process evaluation was conducted alongside the two trials to a) allow better understanding of the COPD and asthma care delivery experiences, for example, feasibility and acceptability for both facility staff and patients; b) distinguish between the care practice deviation because of a faulty design or implementation failure;^12^ and c) inform future scaling on the intervention through public programme resources.

The main aims were therefore to explore how the intended intervention components were implemented and experienced by the care providers and patients; and to identify how the intervention components could be further strengthened for delivering quality chronic respiratory disease care at public health facilities.

## Method

The process evaluation employed a sequential mixed-methods design. The quantitative data from patient clinical records were used to assess the fidelity to the care protocols. The qualitative interviews of service providers and patients were conducted to understand participants' experiences and reasons for deviations from the care protocols. The research team, in consultation with programme staff, identified three sets of task categories to study the intervention. These pre-defined care task categories were: identification and diagnosis of cases; treatment and prevention of chronic lung conditions; and patient follow-up and referral care. Then for each task category, a set of quantitative and qualitative indicators were selected. Box 2 summarises the key selected indicators for the three task categories.

**Box 2.** Selected care tasks and key indicators

Care task	Key indicators	
	Quantitative	Qualitative
Identification and diagnosis of cases	1. Number of asthma and COPD patients registered (of overall outpatient attendance) 2. Number and percentage of asthma and COPD patients receiving PEFR and/or spirometry; and findings thereof (at baseline)	Patient’s and provider’s experiences of (also practice deviations and reasons for): identifying symptoms conducting clinical examination and diagnosis
Treatment and prevention	3. Number and percentage of asthma and COPD patients prescribed inhalers (as per guidelines) and/or other treatment to relieve and/or prevent airway obstruction 4. Smoking cessation rate (comparing baseline and endline status)	Patient’s and provider’s experiences of (also practice deviations and reasons for): prescribing, as per guide dispensing inhalers (drugs) counselling patient (tool-assisted) for smoking cessation coping with input gaps
Patient follow-up and adherence	5. Number and percentage of patient attrition on each follow-up visit (in first 6 months) 6. Number and percentage of patients referred for expert check-up and/or complication and/or severe drug reaction	Patient’s and provider’s experiences of (also practice deviations and reasons for): patient adherence to follow-up visits (include retrieval) staff adherence to care during follow-up visit

COPD = chronic obstructive pulmonary disease. PEFR = peak expiratory flow rate.

The quality of clinical records (the source of quantitative data) was ensured by providing additional training to providers, monitoring the data completeness, and querying any data that appeared implausible. The patient records, collected from July 2015–September 2016, were entered into SPSS (version 17.0). Data were single-entered and the quality was assured by data entry checking at regular intervals to minimise error rates.^13^ The frequency distribution of the data was analysed and cross-tabulated it by sex, and intervention and control.

### Facility and participant selection

The enhanced care (with these products) was implemented and evaluated through a cluster randomised controlled trial at 30 public-funded facilities in three selected districts of Sargodha, Kasur, and Mandi Bahauddin. These facilities were randomised to the intervention and control arm on a 1:1 basis using a lottery method. All patients newly diagnosed with COPD or asthma, aged ≥18 years, and currently living (and expected to continue living for the next 12 months) in the catchment area of the respective health facility were eligible for the study. Patients fulfilling the inclusion criteria but refusing to participate were excluded. A total of 451 asthma and 313 COPD patients were diagnosed, registered, treated, and followed up for ≥6 months by doctors to measure and compare the treatment outcomes (for asthma, control achieved as per Global Initiative for Asthma guidelines; for COPD, BODE index score 0–2) in the intervention and control groups. The results of the COPD trial are forthcoming this journal (*BJGP Open* 2019; in press) and those of the asthma trial are to be submitted for publication; in brief, the interventions were found effective in achieving disease control among registered COPD and asthma patients (see Box 3 for an outline of the intervention and control care).

**Box 3.** Intervention outline

**Tasks** Diagnose on the basis of history and clinical examination Register patient (complete chronic disease card) 1. Prescribe drugs, according to the disease condition (as per desk-guide) **Asthma** Use short acting inhaled beta-2 agonist salbutamol (SABA) for quick relief (as required) Administer beclomethasone inhaler twice a day Change to beclomethasone + formeterol combination if symptoms remain uncontrolled Add montelukast or theophylline if symptoms remain uncontrolled Add oral steroid if symptoms remain uncontrolled **COPD** Start with SABA or short acting muscarine antagonist (SAMA) inhaler Change to SABA and SAMA combination if symptoms remain uncontrolled Change to long acting beta-2 agonist (LABA) + steroid combination if symptoms remain uncontrolled Add long-acting muscarine antagonist LAMA or oral theophylline to LABA + steroid combination if symptoms remain uncontrolled 2. Counsel the patient, using a pictorial tool, for prevention and treatment adherence 3. Examine (clinical), prescribe (and dispense), and record, as per desk-guide, on each monthly follow-up visit 4. Identify and retrieve those found with a delay in their monthly follow-up visit (three-tray system to identify; and a mobile phone to communicate with such patients)

The two tasks (a and b) were common for all asthma and COPD patients. The four tasks (1–4) differed in the intervention and control arms (as per desk-guide in the intervention, and as per doctor routine in the control). LABA = long acting beta-2 agonist. LAMA = long-acting muscarine antagonist. SABA =short-acting inhaled beta-2 agonist. SAMA = short-acting muscarine antagonist.

**Figure 1.**
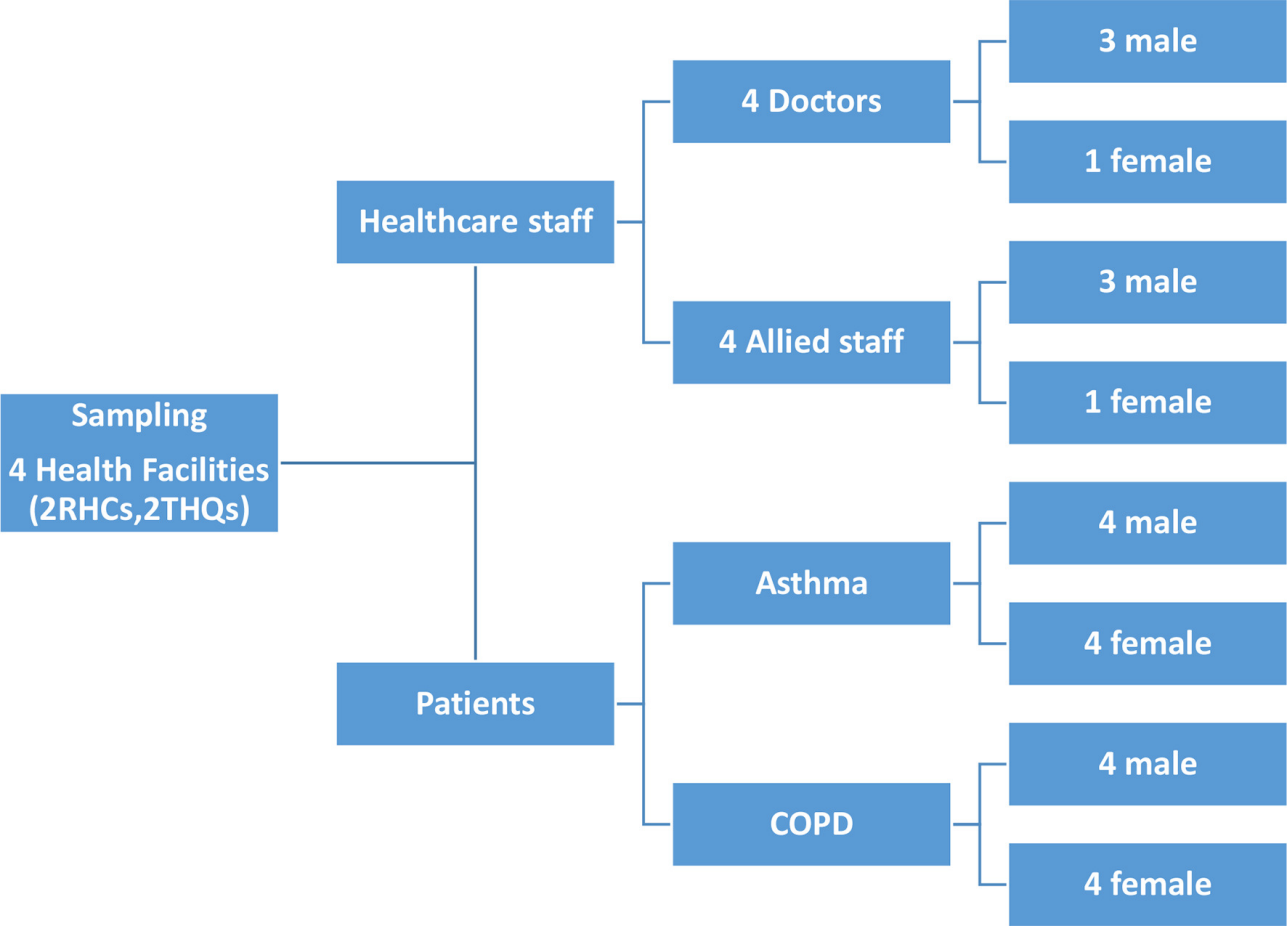
Sampling for interviews COPD = chronic obstructive pulmonary disease. RHC = rural health centre. THQ = Tehsil headquarters.

The quantitative data of all COPD (*n* = 313) and asthma (*n* = 451) patients registered at 30 participating facilities were analysed. To interview the care providers and patients, two districts were randomly selected (Mandi Bahauddin and Kasur); and one rural health centre and one sub-district hospital were randomly selected in each of the two districts. An equal number of rural health centres to sub-district hospitals were purposively selected to get a balanced representation of primary and secondary level facilities. At each of the selected facilities, one doctor (total *n* = 4) and one allied staff member (total *n* = 4)with experience of the intervention were interviewed. In addition, one male and one female patient with COPD (total male COPD *n* = 4; total female COPD *n* = 4), and one male and one female patient with asthma (total male asthma *n* = 4; total female asthma *n* = 4) was randomly selected from registered patients, invited, and interviewed at the respective health facility (see Figure 1 for sampling for interviews).

After the authors' experience in other similar studies,^14,15^ Skype call technology was used by a qualified female researcher to conduct staff and patient interviews, during April–July 2016, at respective facilities. After consent, the interviews were conducted in the national language (Urdu) and were audio-recorded. Each interview, lasting for about 30 minutes, was conducted in a segregated space and the interviewee was assured that their data would be kept confidential and anonymous. A research assistant remained available to assist interviewees with the Skype technology. Three responders were re-contacted by telephone, to elaborate on their initial comments or on responses that were not adequately understood.

The researcher who collected data then listened to the recorded Skype call and transcribed the data. Another researcher then checked the transcripts while listening to the recording. The transcripts were anonymised and the identification keys were kept separate from the data. The coding frame was generated by, firstly, treating the three task categories as a priori overarching themes, and, secondly, two researchers reviewing the transcripts to explore emergent codes and sub-themes. The research team applied the analytical framework to the transcribed data, and responses were charted into the framework matrix.^16^ The framework approach is an analytic process of recognising and managing qualitative data with summarisation to develop a conceptual framework.^17^

The data from different sources (staff and patients) were triangulated, and the key findings and quotations were checked against the original transcripts to ensure trustworthiness and consistency. Ongoing discussion and agreement by the research team framed the analysis process.

## Results

The key findings, from quantitative and qualitative data sets, in each of the three task categories are presented below.

### Identification and diagnosis of chronic respiratory disease

At public-funded facilities, the proposed screening and diagnosis protocol (see Box 4) was found able to diagnose <0.04% of the general outpatient attendees with chronic respiratory disease. In light of the estimated disease prevalence of asthma (4%) and COPD (1%) in the general population, the outpatient attendance seems to indicate relatively low utilisation of public health facilities for chronic lung health care. Interviews with facility staff and patients indicate lower preference of patients with chronic lung condition to seek treatment from a public facility, mainly because of general non-availability of inhalers (or other drugs for chronic respiratory disease) at public facilities.

**Box 4.** An outline of screening and diagnosis protocols for asthma and COPD (extracted from case management desk-guide)

	Asthma	COPD
**Screening**	Asthma is indicated, if: younger patient (though can be an older adult) patient and/or family has history of asthma, allergic rhinitis (hay fever), or eczema patient complains of: recurrent episodes of dry cough and/or difficulty breathing, more so at night or in the morning worsening with exercise, cold, dust, seasonal allergens, or drugs	COPD is indicated, if: middle-aged or older adult who smokes or used to smoke patient has a history of recurrent chest infection patient complains of: progressive persistent shortness of breath (rather than episodic) cough (productive and persistent) exercise worsening the symptoms.
**Assess for asthma or COPD diagnosis, if one or more of the above indications.**		
**Diagnosis**	Diagnose asthma, if patient has history of ≥1 asthma indications, and during an exacerbation has: wheeze (widespread and more on expiration) on investigation (may be normal): PEFR during an exacerbation <80% which improves with bronchodilator other supporting/ indicative investigations: blood CP (eosinophil >5%, though also in bronchitis and COPD) chest X-rays (not usually indicated; may be normal, may be hyperinflation)	Diagnose COPD, if patient has history of ≥1 COPD indications, and has: wheeze – widespread and more on expiration on investigation: PEFR during an exacerbation <80%, with minor or no change with bronchodilator other supporting/ indicative investigations: blood CP (to check for anemia and polycythemia, if required) chest X-rays (vertical heart, hyperinflated lungs, low-set diaphragm)

COPD = chronic obstructive pulmonary disease. Blood CP = blood complete picture. PEFR = peak expiratory flow rate.

As per peak expiratory flow rate (PEFR) measurements, around two-thirds of male and more than half of female (55%) patients had severe airway obstruction when first examined for the diagnosis of asthma and COPD. The response to bronchodilators, measured by PEFR, was found to have been used and reported for all patients to differentiate between asthma and COPD. In asthma, mean PEFR change with bronchodilator was found to be 100 (390–290) in males and 80 (325–245) in females; whereas in COPD the pre-post difference was around 20 in both males and females. Review of individual patient records showed a reasonable level of concurrence between PEFR results and clinical diagnosis (decision). Interviews with the facility staff and registered patients indicate PEFR testing to be feasible for staff, subject to availability of supplies, and acceptable to patients.

As per spirometry, FEV_1_% (forced expiratory lung volume in 1 second as a percent of the predicted amount from a person of similar characteristics) done within 2 weeks of patient diagnosis and registration, around one-third of male (36%), and one-fifth of female (21%) patients had severe airway obstruction. Interviews with the facility and project staff indicated difficulty of offering 'on the spot' spirometry at the time of diagnosis and patient registration at primary and secondary level public facilities, mainly for technical and logistical reasons:

*'We lack equipment and skills for administering spirometry* [on the spot]*; and sending patients to the district hospital* [for spirometry] *was not always possible.'* (Doctor, Sub-District Hospital, Mandi Bahauddin)

The spirometry could only be managed by adding an additional input, for example, a qualified person periodically visiting and conducting spirometry at the respective facilities. The spirometry coverage indicates that most patients were able to pay an extra visit to the facility on a given date and time to receive their spirometry free of charge.

As no other symptoms or signs (such as breathing rate, breathlessness, wheeze, difficulty talking) were required to be recorded in the chronic disease card, it was not possible to comment on clinical practices related to these symptoms. However, most patients mentioned auscultation as part of their clinical examination.

The core demographic data (age, sex); contact details (address, phone number); and clinical parameters (weight, height) were found to have been examined and recorded in all patients registered with a chronic lung condition. The data shows that around two-thirds (*n* = 479, 63%) of the registered chronic lung condition cases were in male patients, and two-thirds (*n* = 316, 66%) of the male patients were aged >45 years. Interviews with facility and project staff revealed that maintaining a patient's chronic disease card is manageable for the regular allied staff at public facilities. However, the facility staff did require supervisory support from the district and/or project staff.

### Treatment and prevention of chronic respiratory diseases

COPD prescriptions at the time of registration showed that, among 159 intervention arm patients, 96 (60%) were prescribed salbutamol, 49 (31%) were prescribed SAMA, and 14 (9%) were prescribed both drugs. Among 154 control arm patients, 133 (86%) were prescribed salbutamol, 18 (12%) were prescribed SAMA, and 3 (2%) were prescribed both drugs. Antibiotics were included in 29 (18%) of intervention arm prescriptions, and 8 (5%) of the control arm prescriptions. Glucocorticosteroid and leukotriene modifier were also included, respectively, in 3 (2%) and 8 (5%) of the intervention arm prescriptions, and 16 (10%) and 32 (21%) of the control arm prescriptions.

Asthma prescription, at the time of registration, showed that among 226 intervention arm patients, 52 (23%) were prescribed salbutamol alone, 72 (32%) were prescribed glucocorticosteroid inhaler alone, and 102 (45%) were prescribed both drugs. Among 225 control arm patients, 115 (51%) were prescribed salbutamol alone, 54 (24%) were prescribed glucocorticosteroid inhaler alone, and 56 (25%) were prescribed both drugs. Leukotriene modifier tablets were included in 23 (10%) of intervention arm prescriptions, and 47 (21%) of the control arm prescriptions. Antibiotics were included in 21 (9%) of intervention arm prescriptions, and 18 (8%) of the control arm prescriptions.

Interviews with facility staff revealed that none of the inhalers could be arranged through district public budget, because of its non-inclusion in the list of essential drugs for the respective facilities:

*'We cannot purchase inhalers from the facility budget, unless these get included in our list of essential drugs.'* (Doctor, Sub-District Hospital, Kasur)

The staff at intervention facilities reported that salbutamol, glucocorticosteroid, and SAMA inhalers were provided through project inputs for free of charge dispensing, whereas leukotriene modifier tablets were prescribed for the patient to buy themselves. No staff member or patient reported any complaint regarding quality of the drugs procured and distributed through the respective programme support.

The facility staff indicated general patient expectation of getting free drugs at a public facility. The non-provision of free drugs (inhalers) seems to add to the risk of patient non-adherence, especially if they are less able to buy drugs. The staff perceives that non-provision of inhalers at public facilities need be addressed before possible scaling of chronic lung healthcare:

*'Free provision of inhalers* [from the health facility] *made it possible for me to take treatment without interruption.'* (Patient, Rural Health Centre, Mandi Bahauddin)

At the time of registration, around one-third of male patients (*n* = 174, 36%), and only 13 (5%) female patients were reported to smoke. At the time of outcome measurement after 6 months of registration, *n* = 92/174 (53%) male patients and *n* = 1/13 (7%) female patients were reported to have continued smoking. The reported average duration of the first smoking cessation counselling session was about 15 minutes, whereas duration of subsequent counselling sessions was generally <5 minutes. Staff and patients reported that members of staff prioritised smoking cessation counselling for patients with chronic lung conditions, and neither faced logistical or social constraint to the patient being counseled:

*“Patients with chronic lung conditions are generally more receptive to our 'quit smoking' advice.”* (Paramedic, Sub-District Hospital, Kasur)

### Patient follow-up and adherence

**Figure 2.**
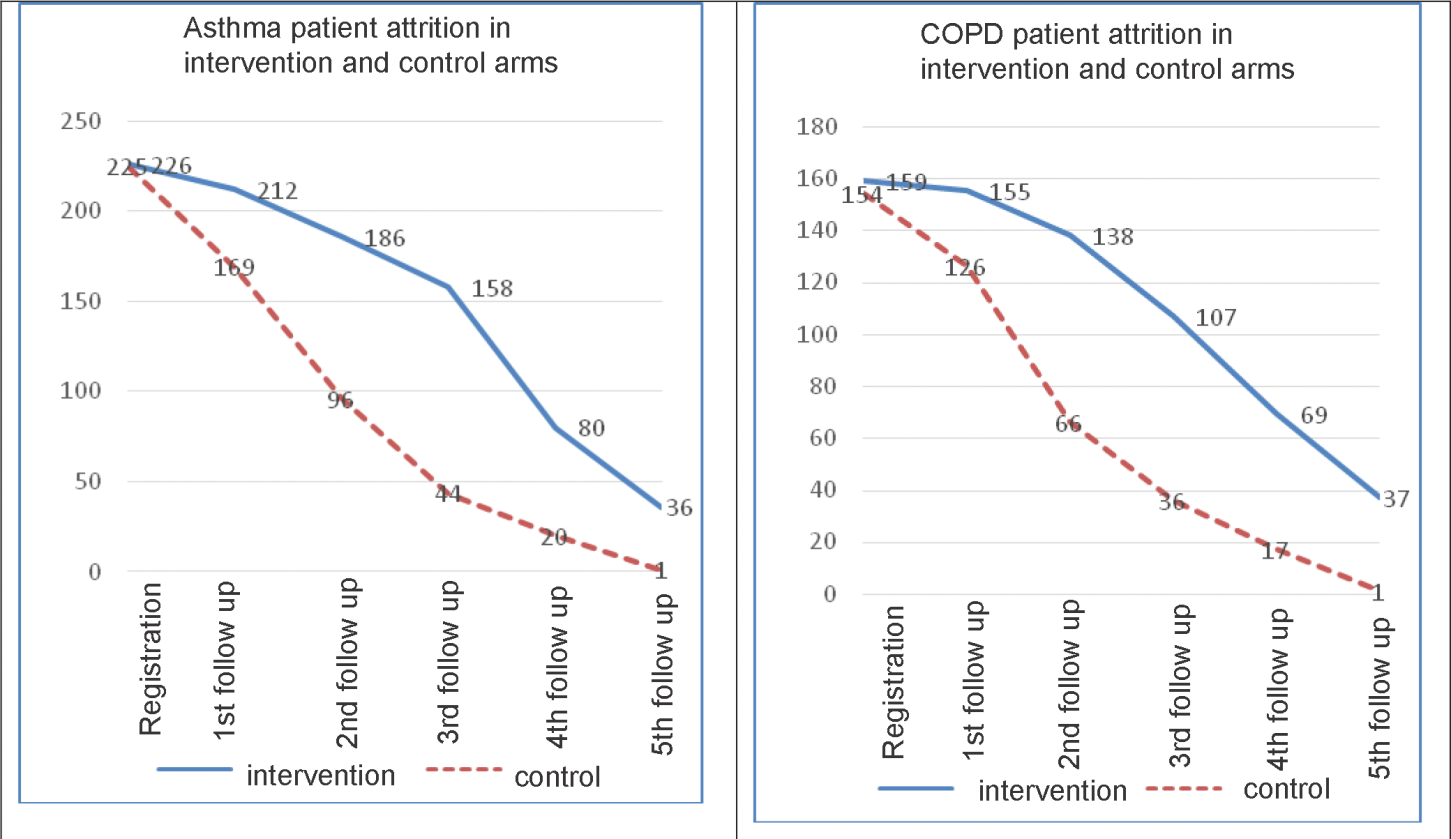
Asthma and COPD patient attrition in intervention and control arms COPD = chronic obstructive pulmonary disease.

As shown in Figure 2, the adherence rate for the first five follow-up visits in COPD and asthma patients was 64% (*n* = 506/795) and 60% (*n* = 672/1130) respectively in the intervention arm, compared to 32% (*n* = 246/770) and 30% (*n* = 330/1125) respectively in the control arm. Patient non-adherence to follow-up visits during the first 6 months seems to be a major treatment challenge in both the intervention and control arms. However, comparison of the two arms indicates relatively lower and slower attrition among those exposed to the intervention. This difference seems more marked for the second and third follow-up visits. The three-tray system was reported mostly to be in place for managing patient cards at 15 intervention health facilities. Interviews with staff and patients indicated that free drugs were an incentive for patients to attend the facility, and that enhanced patient retrieval was helpful for adherence to follow-up visits. The allied staff reported no difficulties with regards calling to remind male and female patients about their follow-up visits:

*'I generally use the mobile number of husbands to remind women* [patients] *about their due follow-up visits.'* (Paramedic, Rural Health Centre, Kasur)

The record review of 576 (intervention COPD and asthma combined) and 1178 (control COPD and asthma combined) follow-up visits, showed 98% compliance (*n* = 1719/1754) to the assessment and recording of PEFR at each follow-up visit. The staff did not report any challenge to examining the patient on their follow-up visits. However, the staff did report the importance of uninterrupted supplies (such as disposable mouth pieces) and equipment maintenance (for example, of the peak flow meter) for their compliance with the patient examination requirement. Some patients seem to face social challenges to accessing free lung care (including drugs) at public funded facilities:

*'Some male patients find it difficult to come every month, because of other engagements during the hospital* [outpatient] *timing.'* (Paramedic, Sub-District Hospital, Mandi Bhauddin)

The referral of patients with severe lung conditions was reported to have been practised at public facilities, but no records of this were found to have been maintained. The facility staff indicated that patient families appraise the referral care suggestion, and select the source and the timing of specialist care (when advised) in light of the advice and their own circumstances.

## Discussion

### Summary

The two cluster randomised trials have shown the integrated care at primary and secondary level health facilities to be effective in controlling two chronic lung conditions (COPD and asthma). This process evaluation study showed that the delivery of integrated chronic lung care, as per programme protocols, is feasible for staff and patients in routine PHC settings.

### Strengths and limitations

Using routine clinical records as the main source of quantitative analysis made the data collection efficient (for example, causing minimal added cost), but some variables of interest were not available, such as education level and occupation. There is a lack of literature evaluating the use of Skype technology to interview health workers and patients in applied health research, with participant discomfort and technical failure possible drawbacks. The authors' experience, however, was broadly positive, and use of Skype technology enabled the more efficient use of the expert interviewer’s time.

### Comparison with existing literature

The rate of reported smoking cessation by male patients with chronic lung condition (47%), was close to the cessation rate previously achieved (around 40%) among patients with suspected tuberculosis in Pakistan.^8^ The intervention was found to have improved the attendance for follow-up visits. However, high attrition (even in intervention arm) does indicate the need for improving treatment adherence through implementation research on various potential options,^18^ such as reducing the frequency of follow-up visits (unless clinically indicated),^14^ extending the timing of hospital outpatient, adding a treatment supporter,^19^ and developing public–private partnership for expanded coverage and continued care.^20^

To assess the degree of airway obstruction at the time of diagnosis or registration, healthcare staff performed PEFR examination on the spot, and spirometry was administered, as a research related measurement, within 2 weeks of patient diagnosis. In this study, spirometry (as compared to PEFR) identified a lower number (proportion) of severe airway obstruction among registered patients. This is different from other reported experience,^21^ which found that spirometry to be a more sensitive measure of airway obstruction severity. As spirometry (for logistic reasons) was administered within 2 weeks of treatment initiation, spirometry identifying lower number (proportion) of severe airway obstructions in this study seems plausible.

This study identified both equipment and staff skill challenges to offering on the spot spirometry at primary and secondary level health facilities in a developing country setting. Experiences in two developed countries (UK^22^ and Italy)^23^ found offering spirometry without specialist input to be feasible, but managing providers’ protocol violation and patients’ inability to participate or perform did pose challenges. Furthermore, the UK study found spirometry to add to the quality of diagnosis at general practice level; in Italy the evidence for the same was inconclusive. The present study's findings do not suggest that decentralising spirometry to the PHC level would be feasible.

Availability of drugs is considered important for patients to attend their facility and adhere to treatment.^24^ At the time when the trial was implemented, inhalers (the main drug used to treat chronic lung conditions) were not included in the list of essential drugs for PHC facilities. Inhalers were included as part of the intervention arm package to generate experience-based learning for scaled implementation. Education on how to administer inhalers, as an important care component,^25^ was also made a part of patient counselling at registration and during follow-up visits.

About 20% of asthma and COPD patients being prescribed leukotriene modifier tablets seems in line with the generally reported^26^ tendency of physicians to overprescribe, even when the drug is not made available for free dispensing. Another study^14^ on non-communicable disease care in Pakistan reported the prescription of drugs to be a blend of doctor and patient preferences, with some doctors prescribing high-cost pharmaceutical products because of fancy packaging and active marketing.

### Implications for research and practice

In light of effectiveness (as shown in the two trial results) and feasibility (as shown by the process evaluation study), the integrated chronic lung care intervention is being scaled in the province, and inhalers have also been added to the list of essential drugs for PHC facilities. However, implementation research may be considered for enhanced adherence to follow-up care.

This study shows that integrating COPD and asthma care into routine healthcare at primary and secondary level public facilities is feasible and acceptable. Integrated COPD and asthma care can lead to improved assessment, diagnosis, prescription practices, and adherence to follow-up appointments in a low-income country setting, with promising results. The evidence from the trial and the process evaluation study support the recommendation for scaled implementation in developing country settings.
